# Aberrant resting-state brain activity in Huntington's disease: A voxel-based meta-analysis

**DOI:** 10.3389/fneur.2023.1124158

**Published:** 2023-03-30

**Authors:** Sirui Zhang, Junyu Lin, Yangfan Cheng, Yanbin Hou, Huifang Shang

**Affiliations:** ^1^Department of Neurology, West China Hospital, Rare Disease Center, Sichuan University, Chengdu, China; ^2^Laboratory of Neurodegenerative Disorders, National Clinical Research Center for Geriatric, West China Hospital, Sichuan University, Chengdu, China; ^3^West China School of Medicine, West China Hospital, Sichuan University, Chengdu, China

**Keywords:** Huntington's disease, meta-analysis, resting-state, functional magnetic resonance imaging, psychoradiology

## Abstract

**Introduction:**

Functional neuroimaging could provide abundant information of underling pathophysiological mechanisms of the clinical triad including motor, cognitive and psychiatric impairment in Huntington's Disease (HD).

**Methods:**

We performed a voxel-based meta-analysis using anisotropic effect size-signed differential mapping (AES-SDM) method.

**Results:**

6 studies (78 symptomatic HD, 102 premanifest HD and 131 healthy controls) were included in total. Altered resting-state brain activity was primarily detected in the bilateral medial part of superior frontal gyrus, bilateral anterior cingulate/paracingulate gyrus, left insula, left striatum, right cortico-spinal projections area, right inferior temporal gyrus area, right thalamus, right cerebellum and right gyrus rectus area. Premanifest and symptomatic HD patients showed different alterative pattern in the subgroup analyses.

**Discussion:**

The robust and consistent abnormalities in the specific brain regions identified in the current study could help to understand the pathophysiology of HD and explore reliable neuroimaging biomarkers for monitoring disease progression, or even predicting the onset of premanifest HD patients.

## Introduction

Huntington's disease (HD) is a hereditary neurodegenerative disease caused by cytosine–adenine–guanine (CAG) repeat expansion in the first exon of the huntingtin (HTT) gene on chromosome 4, mainly affecting the striatum and the cortex progressively with disease development (1, 2). The identification of optimal, robust, and early biomarkers are important and can reflect disease progression and response to treatment, especially for mutation carriers in the premanifest stage of HD with no obvious clinical manifestations. The functional neuroimaging method provides new insights.

Compared with brain structure atrophy, functional imaging alterations could be detected in the early stage and considered more sensitive as biomarkers (3). In addition, functional imaging may provide abundant information on the underlying pathophysiological mechanisms of the clinical triad including motor, cognitive, and psychiatric abnormalities in HD (3, 4). The blood oxygen level-dependent (BOLD) signal of resting-state fMRI (rs-fMRI) indirectly reflects regional brain activity, and several approaches have been utilized to analyze spontaneous BOLD signals including the amplitude of low-frequency oscillations (ALFF), regional homogeneity (ReHo), and independent component analysis (ICA) (5). Other radiotracer techniques measuring regional cerebral blood flow (rCBF) or glucose metabolism (rCMglu) can also detect neuronal activity (5). Existing studies using these methods to reflect aberrant brain activity in HD demonstrated inconsistent results (6–11), and there has been no quantitative meta-analysis performed to date (3).

We aimed to identify the most consistent and replicable regions demonstrating abnormal intrinsic brain activity among HD patients compared with healthy controls (HCs) and expected to detect different patterns of alterations among premanifest and manifest HD patients separately in the subgroup analyses. This might be the first study to portray resting-state brain activity abnormalities in HD patients using the method of a voxel-based meta-analysis.

## Materials and methods

### Literature search

The comprehensive literature search was performed in the MEDLINE, EMBASE, and Web of Science databases following the Preferred Reporting Items for Systematic Reviews and Meta-Analyses (PRISMA) guidelines strictly (12). Two independent investigators (ZSR and LJY) searched all available and relevant studies from database inception to 10 November 2022. Manual searches were also conducted within the reference list of identified articles to avoid omission and supplement the initial search. As an example, the detailed search strategy in MEDLINE is presented in Supplementary Table 1.

### Study selection and data extraction

The inclusion criteria for the current meta-analysis were as follows: studies that (1) included premanifest or manifest genetically confirmed HD mutation carriers and healthy controls; (2) employed one of the functional imaging methods such as fMRI, PET, and SPECT in the resting state; (3) applied a whole-brain analysis and used a significant and consistent threshold throughout the whole brain; (4) reported coordinates of the abnormal brain activity in a standard stereotactic space [Talairach or Montreal Neurological Institute (MNI)] or detected no significant differences. Studies were excluded if (1) studies used seed-based analysis procedures or limited the analysis to a specific region of interest, (2) studies were review articles and case reports reporting no original data, and (3) studies were conference abstracts without available full articles and relevant data. In the case that two studies were performed based on overlapping patient cohorts, the study with a smaller sample size would be excluded. A total of two investigators (SZ and JL) performed the study selection independently. Any discrepancies that could not be resolved through discussion were addressed by the third author (HS).

After literature selection, we extracted the following information and data in each included study: first author, publication year, country, sample size, baseline demographic information, clinical characteristics of included participants, software, peak coordinates, and the corresponding effect sizes (*t*-values, *p*-values, and Z-scores). *p*-values and Z-scores were converted to *t*-values for analyses using the SDM online converter (https://www.sdmproject.com/utilities/?show=Statistics). In cases in which the studies did not report any forms of the effect size, we wrote a “*p*” for positive peaks and “*n*” for negative peaks following the AES-SDM software package guidelines.

### Quality assessment

At the time the study was conducted, there was no standard quality assessment checklist for voxel-based meta-analyses. Following those described in previous meta-analyses (5, 13), a 10-point quality assessment checklist was applied in the current study (Supplementary Table 2). This checklist evaluated the quality of studies in terms of three aspects: sample characteristics, methods for image acquisition and analysis, and results reporting. Studies yielded a 0/0.5/1 score for each item (0 representing not met, 0.5 representing partly met, and 1 representing completely met).

### Statistical analysis

AES-SDM software was used to perform the statistical analysis following the AES-SDM tutorial and “Ten simple rules for neuroimaging meta-analysis”(14), which has been used to investigate the neural substrates of psychological functions or some other neuropsychiatric disorders (15, 16). The detailed algorithm and theory of this software were elaborated in the previous literature (17–19).

### Pooled meta-analysis

Statistical parametric maps of individual studies were first recreated for the pre-processing of peak coordinates using the method of an anisotropic un-normalized Gaussian kernel (18). The mean of the voxel values in different studies was weighted by the inverse of the variance and accounted for inter-study heterogeneity (17). The detailed method and its advantages were described elsewhere (17, 19). The recommended parameters [full width at half maximum (FWHM) = 20 mm, *p* = 0.005, peak height threshold = 1, and extent threshold = 100 voxels] were applied in the analyses (17, 20). The results were presented using MRIcron software. The potential effect of age and CAG repeats number on the results of our analyses were examined using meta-regression analysis, and a conservative threshold of a *p*-value of < 0.0005 was applied. The subgroup analyses of premanifest HD patients (pHD) and symptomatic HD patients (sHD) were performed separately.

### Sensitivity analysis

Jackknife sensitivity analysis was performed to test the robustness of the results by repeating the meta-analysis procedure multiple times and removing a single study from the analysis each time. If previously identified regions of abnormal alterations remained significant in all or most combinations of studies, we considered results replicable and stable.

### Heterogeneity analysis and publication bias

Between-study heterogeneity was assessed using a random-effects model with Q statistics transformed to SPM-Z values. Publication bias was examined using funnel plots and Egger's test. An obvious asymmetric funnel plot and a *p*-value of < 0.05 for Egger's test suggested evidence for the presence of publication bias (21).

## Results

### Included studies and sample characteristics

**Figure 1:**
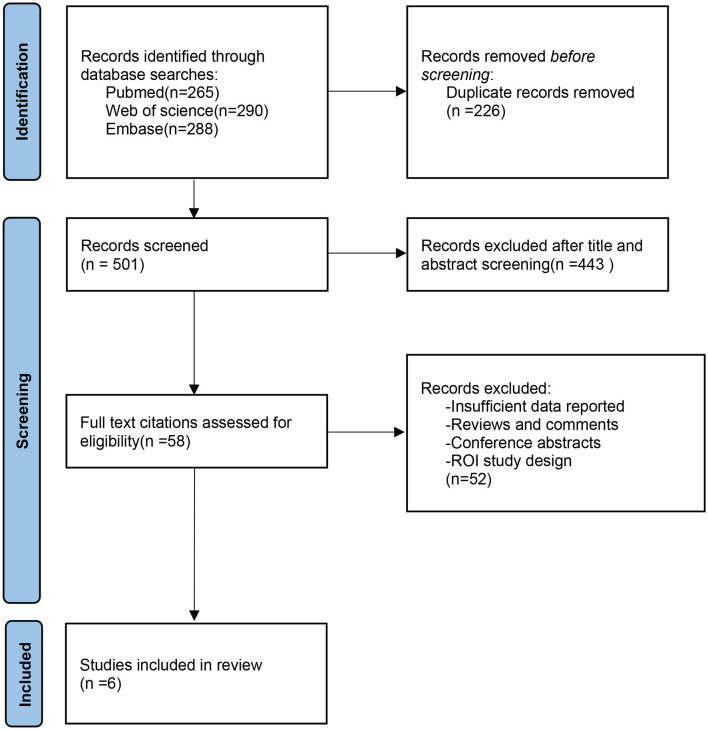
PRISMA flow diagram representing screening and selection procedure in meta-analysis.

Among the 843 studies identified in our searches, we included six studies that met the inclusion criteria. The detailed process of study screening and selection is presented in the PRISMA flow diagram (Figure 1). Of the six included studies, five studies investigated abnormal intrinsic brain activities using the method of rs-fMRI (7–11), while only one study assessed the change of brain activities reflected by resting-state cerebral blood flow (rs-CBF) using the magnetic resonance perfusion imaging method (6). A total of two studies compared pHD patients with healthy controls (HCs) (6, 10), two studies compared sHD with HCs (7, 9), and two studies compared both pHD and sHD patients with HC separately (8, 11). The rs-fMRI study by Sarappa et al. analyzed both fALFF and ReHO abnormality of premanifest and manifest HD patients and was considered two independent datasets (11). A total of six included studies provided 10 datasets for the analyses. A pooled population of 78 sHD, 102 pHD, and 131 HC was included. Table 1 summarizes the detailed demographic information and clinical characteristics of the included studies. The quality scores ranged from 8.5 to 9.5, indicating the moderate-to-high quality of the included studies and are summarized in Supplementary Table 3.

**Table 1:** Detailed characteristics of each study.

**Study**	**Country**	**Modality/analysis**	**Diagnosis**	**Disease stage**	**Sample number**			**Mean Age** ±**SD**			**Male/Female**		
					**pHD**	**sHD**	**HC**	**pHD**	**sHD**	**HC**	**pHD**	**sHD**	**HC**
Wolf et al. (6)	Germany	MRI (3T,CASL)/rCBF	Gene testing	pHD	18	NA	18	36.3 ± 9.0	NA	37.2 ± 10.3	8/10	NA	9/9
Werner et al. (7)	Germany	rs-fMRI (3T)/ICA	Gene testing (CAG repeat expansion 40-49)	sHD	NA	17	19	NA	44.9 ± 9.9	47.5 ± 10.1	NA	7/10	8/11
Poudel et al. (8)	Australia	rs-fMRI (3T)/ICA	Gene testing	pHD&sHD	25	23	18	42.9 ± 9.2	56.0 ± 9.4	45.5 ± 13.7	9/16	13/10	4/14
Liu et al. (9)	China	rs-fMRI (3T)/ALFF	Gene testing	sHD	NA	10	20	NA	45.0 ± 9.1	45.4 ± 8.4	NA	1/9	2/18
Sarappa et al. (11)	Italy	rs-fMRI (3T)/fALFF, ReHo	Gene testing	pHD&sHD	11	28	40	38.1 ± 7.1	41.6 ± 9.6	37.4 ± 13.5	5/6	17/11	18/22
Harrington et al. (10)	USA	rs-fMRI (3T)/NBS	Gene testing	pHD	48	NA	16	39.7 ± 10.4	NA	42.6 ± 9.2	8/40	NA	4/12
**Education years**			**CAG repeats number**		**UHDRS motor score**			**UHDRS cognitive score**			**Software**	**Atrophy correction**
**pHD**	**sHD**	**HC**	**pHD**	**sHD**	**pHD**	**sHD**	**HC**	**pHD**	**sHD**	**HC**		
14.7 ± 2.0	NA	15.1 ± 2.7	42.1 ± 3.1	NA	3.1 ± 3.0	NA	NA	329.8 ± 32.3	NA	NA	SPM8	No
NA	NA	NA	NA	44.2 ± 2.6	NA	31.1 ± 20.2	1.0 ± 1.0	NA	208.7 ± 81.1	319.3 ± 35.9	MELODIC, FSL	Yes
NA	NA	NA	42.5 ± 1.9	42.6 ± 2.0	1.0 ± 1.2	26.5 ± 18.2	NA	NA	NA	NA	SPM8, FSL	No
NA	10.4 ± 2.76	NA	NA	43.9 ± 4.2	NA	25.7 ± 15.2	NA	NA	NA	NA	SPM8	Yes
NA	NA	NA	46 (range 40–49)	46 (range 42–65)	1 (range 0–4)	17 (range 5–53)	NA	216 (106–324)	166 (range 52–234)	NA	SPM8	Yes
14.5 ± 2.4	NA	16.0 ± 1.9	42.7 ± 2.6	NA	7.3 ± 5.3	NA	5.1 ± 4.5	NA	NA	NA	Not reported	No

CASL, continuous arterial spin labeling; pHD, premanifest HD; sHD, symptomatic HD; HC, healthy control; ALFF, Amplitude of Low Frequency Fluctuations; ReHo, regional homogeneity; ICA, independent component analysis; fALFF, fractional Amplitude of Low Frequency Fluctuations; NBS, network-based statistic; MELODIC, Multivariate Exploratory Linear Decomposition into Independent Components; SPM, Statistical Parametric Mappin; NA, not available.

### Pooled meta-analyses and Jackknife sensitivity analyses

**Figure 2:**
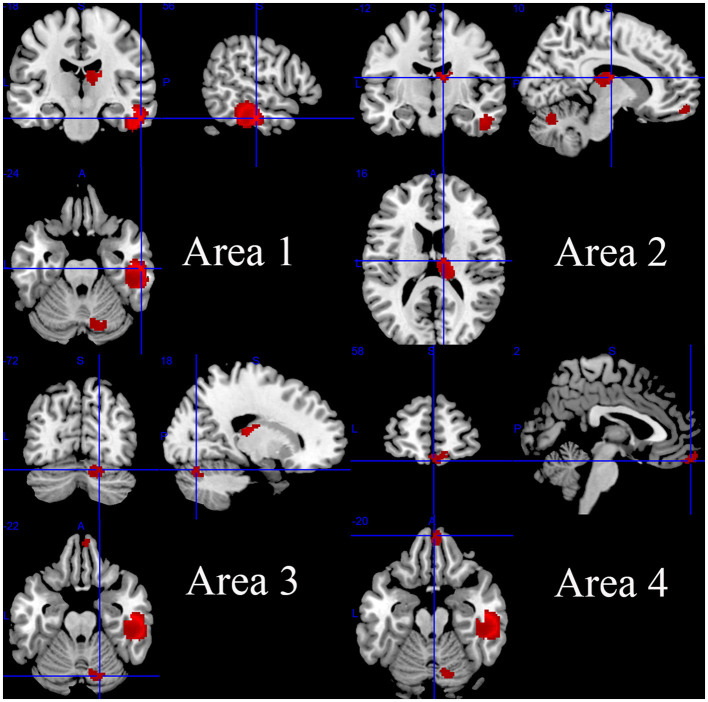
Regions of increased intrinsic resting-state brain activity in patients with HD.

**Figure 3:**
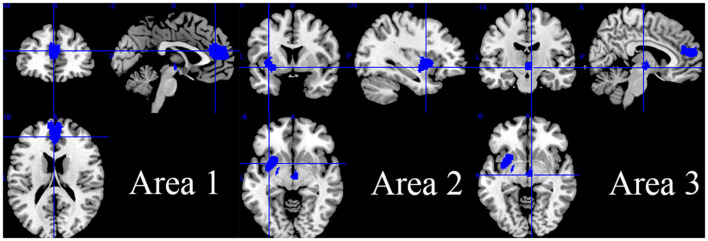
Regions of decreased intrinsic resting-state brain activity in patients with HD.

Compared with the HC group, the HD group including both pHD and sHD showed increased intrinsic resting-state brain activity in the right inferior temporal gyrus, inferior longitudinal fasciculus, right fusiform gyrus, right middle temporal gyrus, right thalamus, right anterior thalamic projections, right cerebellum (hemispheric lobule VI and crus I), right superior frontal gyrus (orbital part), and corpus callosum (Figure 2 and Table 2). The HD group showed decreased intrinsic brain activity in the bilateral superior frontal gyrus (medial part), bilateral anterior cingulate/paracingulate gyri, corpus callosum, left insula, left striatum, left amygdala, anterior commissure, right anterior thalamic projections, and right thalamus (Figure 3 and Table 2). Most findings remained highly or moderately stable and replicable in the Jackknife sensitivity analyses (significant in at least seven combinations), while only six combinations detected a significant decrease in brain activity in the right anterior thalamic projections (Table 2).

**Table 2:** The mean meta-analysis: altered resting-state activity in HD patients relative to HCs.

**Contrast**	**Brain region**	**MNI coordinates**			**SDM-Z score**	**No. of voxels**	***p*-value**	**Egger's test (*p*)**	**Clusters' breakdown**	**Jackknife sensitive analysis**

		**X**	**Y**	**Z**						
HD>HC	Area 1(Right inferior temporal gyrus)	56	−18	−24	2.264	1174	< 0.001	0.153	Right inferior temporal gyrus	10/10
									Right inferior network, inferior longitudinal fasciculus	10/10
									Right fusiform gyrus	10/10
									Right middle temporal gyrus	10/10
									Corpus callosum	10/10
	Area 2 (Right thalamus)	10	−12	16	1.938	277	< 0.001	0.225	Right thalamus	10/10
									Right anterior thalamic projections	10/10
	Area 3 (Right cerebellum, hemispheric lobule VI)	18	−72	−22	1.601	180	0.001	0.269	Right cerebellum, hemispheric lobule VI	9/10
									Right cerebellum, crus I	7/10
	Area 4 (Right gyrus rectus)	2	58	−20	1.507	162	0.002	0.883	Right gyrus rectus	8/10
									Right superior frontal gyrus, medial orbital	8/10
									Right superior frontal gyrus, orbital part	8/10
									Corpus callosum	10/10
HD < HC	Area 1 (Left anterior cingulate / paracingulate gyri)	−2	44	18	−2.566	1103	< 0.001	0.083	Left superior frontal gyrus, medial	10/10
									Left anterior cingulate/paracingulate gyri	10/10
									Right superior frontal gyrus, medial	10/10
									Right anterior cingulate/paracingulate gyri	10/10
									Corpus callosum	10/10
	Area 2 (Left insula)	−34	6	−6	−2.124	616	< 0.001	0.412	Left insula	8/10
									Left lenticular nucleus, putamen	8/10
									Left striatum	7/10
									Left amygdala	7/10
									Anterior commissure	7/10
	Area 3 (Right cortico-spinal projections)	6	−14	−6	−2.011	128	< 0.001	0.377	Right anterior thalamic projections	6/10
									Right thalamus	7/10

### Analyses of heterogeneity and publication bias

Most regions with significant abnormal intrinsic brain activity alterations showed no significant between-study heterogeneity except the right inferior longitudinal fasciculus, right inferior temporal gyrus, and right fusiform gyrus. Both funnel plots (Supplementary Figures 1, 2) and Egger's tests (Table 2) indicated no obvious publication bias.

### Meta-regression analyses

Meta-regression analyses were performed in the HD group and HC group. The regression analyses found that the altered resting-state brain activity was significantly associated with age and CAG repeats number but not with UHDRS motor scores. To be specific, there was an increased probability of finding decreased brain activity in the bilateral superior frontal gyrus (medial part) and bilateral anterior cingulate/paracingulate gyri among younger mutation carriers (*p* < 0.0005). In addition, there was an increased probability of finding increased brain activity in the right inferior temporal gyrus, right inferior longitudinal fasciculus, right fusiform gyrus, and right middle temporal gyrus among those mutation carriers with more CAG repeat numbers (*p* < 0.0005).

### Subgroup meta-analyses and Jackknife sensitivity analyses

The subgroup analyses comparing pHD with HCs detected increased brain activity mainly in the right thalamus area including the right thalamus, right anterior thalamic projections, right caudate nucleus, and corpus callosum, and detected decreased brain activity in the bilateral anterior cingulate/paracingulate gyri, left superior frontal gyrus (medial part), and right anterior thalamic projections. Right anterior thalamic projections were unstable in the Jackknife sensitivity analyses and remained significant only in two combinations (Supplementary Table 4).

Comparing sHD with HCs, sHD patients showed increased brain activity in areas mostly consistent with the main analysis including the right inferior temporal gyrus, right inferior longitudinal fasciculus, right fusiform gyrus, right middle temporal gyrus, corpus callosum, bilateral superior frontal gyrus (medial orbital part), bilateral gyrus rectus, right cerebellum hemispheric lobule VI, and crus I (Supplementary Table 3 and Supplementary Figure 2). sHD patients showed decreased brain activity mainly in the bilateral superior frontal gyrus (medial part), corpus callosum, bilateral anterior cingulate/paracingulate gyri, and right striatum (Supplementary Table 4).

## Discussion

Using the method of AES-SDM meta-analysis, we found that the intrinsic brain activity decreased mainly in the bilateral superior frontal gyrus (medial part), bilateral anterior cingulate/paracingulate gyri, left insula area, and right cortico-spinal projections area and increased mainly in the right inferior temporal gyrus area, right thalamus area, right cerebellum (hemispheric lobule VI) area, and right gyrus rectus area. Mixed alterations of brain activity (both increased coordinates and decreased coordinates were detected within one brain region) were observed in the right superior frontal gyrus (medial part), right thalamus, and corpus callosum.

Previous structural and functional studies have reported that anteromedial superior frontal gyrus (SFG) was connected with the anterior and mid-cingulate cortex as key nodes in the default mode network (DMN) and executive network (ECN) (22) and was also associated with emotion, motivation, and sociability regulation-related processes (23, 24). Decreased brain activity was identified most prominently in the bilateral medial part of SFG and the bilateral anterior cingulate gyrus, indicating the functional loss of DMN and ECN. Consistent with our findings, previous resting-state fMRI studies generally observed abnormal functional connectivity of DMN in HD. The abnormal function of ECN, which exerted important functions in the cognitive domain and might also correlate tightly with a cognitive decline in HD, was widely reported in previous studies (8, 25, 26). As we did not detect other abnormalities of critical components in DMN such as precuneus and angular gyrus (9, 25), the functional alterations of SFG and anterior cingulate/paracingulate gyrus observed in the current meta-analysis might contribute prominently to the abnormal functional connectivity of DMN and ECN and can partially explain some important aspects of the clinical manifestations of HD patients.

Although structural studies of HD have generally found that striatum atrophy is a hallmark of HD patients even in the early stage and functional abnormalities have been considered sensible compared to structural atrophy, the current study only showed moderately decreased brain activity in the left striatum. This is consistent with most of the previous functional neuroimaging studies (27), supporting that the structural atrophy of the striatum did not represent the functional downregulation, and a dissociation pattern of structural and functional alterations exists. This might be partially explained by the neural compensation theory (26). Increased inferior temporal gyrus (ITG) activity was observed in the current meta-analysis, which is involved in the processes of visual object recognition and might be tightly associated with the impaired recognition memory of hand positions and spatial locations in HD patients (9, 28). In addition, ITG also presents an important correlation with the striatum through the temporo-striatal circuit. The structural atrophy and partial functional loss of the striatum might be compensated by the increased activity of ITG to maintain the normal function of the temporo-striatal circuit. Projections from the striatum to the frontal motor regions are also widely investigated (29, 30), and previous studies detected a pattern of alterations similar to the temporo-striatal circuit in the frontal-striatal circuit, which is of great vitality in maintaining executive function (9). However, in the current study, after the meta-analysis of the included studies, only the significantly decreased brain activity of the prefrontal cortex was observed in our analyses, which might indicate that the frontal-striatal circuit is damaged more severely.

The structural imaging studies of HD generally considered the striatum and the cortex as the primary location of pathology, and the cerebellum also showed considerable atrophy in HD (31) and played an important role in HD (31–33). The degeneration of the cerebellum in HD is correlated with disrupted fine motor skills, postural instability, impaired rapid alternating movements, etc (31). Our results also showed that the resting-state intrinsic activity of the right cerebellum increased in HD patients, possibly suggesting that increased neural activity was required to counterbalance the structural atrophy of the cerebellum.

We also found that even within one single brain region, the alteration is complicated and not unidirectional, especially referring to the right thalamus region in the current study. Such divergent alteration patterns were also observed in DMN and ECN in previous studies (3). They detected a functional posterior–anterior dissociation pattern within the ECN in HD (25, 26), potentially representing a compensatory mechanism to counterbalance the downregulation of disrupted brain regions during disease progression (3). The complicated alteration pattern might also be associated with the heterogeneity of included studies and deserved further investigation to explore the role of neural compensation in HD progression and its correlation with clinical manifestations. Interestingly, brain regions with decreased activity in HD were always bilateral, while brain activity always increased on the right side. The underlying mechanism of such asymmetry is still hard to explain, but two cerebral hemispheres with different functions may present different susceptibility or resistance. This finding may also be caused by the between-study heterogeneity and hence should be interpreted with caution. Future studies with larger sample sizes may help to verify the asymmetry.

In the meta-regression analyses, our results showed that younger patients or patients with more CAG repeat numbers were more sensible to detect altered intrinsic brain activity, which might be associated with more severe pathogenic changes and less neural compensation among these patients. Although we reasonably speculated that the observed abnormal resting-state brain activity of the specific brain region was involved in the pathophysiological mechanisms of HD and correlated with patients' manifestations, UHDRS motor scores were not significantly associated with the abnormal resting-state brain activity, and the effect of cognitive performances was not analyzed in the current study limited by incomplete information of original data. A possible reason for the negative finding is that among studies with notable different UHDRS motor scores in the baseline, the results varied widely and were too inconsistent to detect a significant correlation. Wolf et al. reported that the anterior cingulate gyrus within the left lateral prefrontal resting-state network was associated with better motor performances, and higher middle frontal gyrus functional connectivity within the anterior prefrontal resting-state network was associated with better cognitive ability (34). Left ITG neural activity was also reported to be significantly correlated with executive function (9). However, one longitudinal study assessing the functional connectivity changes reported that the functional connectivity did not change significantly over 3 years and lacked sensitivity compared to striatal atrophy (35), suggesting that the validation of altered intrinsic brain activity as functional imaging biomarkers still requires future evidence.

We expected to summarize the dynamic change pattern of the resting state from the subgroup analysis of pHD and sHD separately. We observed that decreased brain activity in the left medial superior frontal gyri and bilateral anterior cingulate/paracingulate gyri started in the pHD stage and maintained dysfunction in the sHD stage. Most regions with increased intrinsic brain activity were only detected in the sHD stage but not in the pHD stage. Future large sample size studies are warranted to further clarify the dynamic change pattern of HD.

The current meta-analysis is a preliminary exploratory study to portray the alteration pattern of the resting-state brain activity in HD patients with several limitations: (1) Only six studies were included in the current meta-analyses, and the sample size of the current study is relatively small. There was also heterogeneity among the studies included. The findings of the current study should be interpreted cautiously. (2) Following the AES-SDM guideline and previous voxel-based meta-analysis (5, 36–38), we included all studies using resting-state neuroimaging methods focused on brain activity which might lead to the heterogeneity between studies considering different physiological bases of different methods. However, in the Jackknife sensitivity analyses, the results remain stable in the combination of rs-fMRI studies. (3) We also excluded all experiments applying the ROI method that may lead to a bias as a critical number of studies may not be considered in the meta-analysis. We hence discussed some important findings of previous ROI studies in the discussion part as recommended in the software guideline (14). (4) The AES-SDM meta-analysis was conducted based on reported results, but not original data, which may affect the accuracy of the identified spatial location.

## Conclusion

In this voxel-based meta-analysis including six resting-state functional neuroimaging studies, we preliminarily portrayed the whole-brain activity alterations in HD. We found that altered resting-state brain activity mainly presented in the bilateral medial part of the superior frontal gyrus, bilateral anterior cingulate/paracingulate gyrus, left insula area, right cortico-spinal projections area, right inferior temporal gyrus area, right thalamus, right cerebellum, and right gyrus rectus area. The pHD and sHD patients showed different patterns of alteration and regions with increased brain activity mostly presented in the sHD stage.

## Data availability statement

The original contributions presented in the study are included in the article/Supplementary material, further inquiries can be directed to the corresponding author.

## Author contributions

SZ contributed to the conception, data collection, statistical analysis, and drafting of the manuscript. JL, YH, and YC contributed to the data collection and drafting of the manuscript. HS contributed to the conception and organization and review of the manuscript. All authors contributed to the article and approved the submitted version.
