# Supplementary material for: Aberrant resting-state brain activity in Huntington's disease: A voxel-based meta-analysis
Source: Front Neurol. 2023 Mar 30;14:1124158. doi: 10.3389/fneur.2023.1124158 (PMC10098104; doi:10.3389/fneur.2023.1124158)
Supplement: Supplementary file 1 [file Data_Sheet_1.docx]

**Supplementary Materials**

**Supplementary Table 1 Detailed search strategy**

| #1 | Huntington’s Disease |
| --- | --- |
| #2 | HD |
| #3 | #1 or #2 |
| #4 | resting-state |
| #5 | rs-fMRI |
| #6 | resting |
| #7 | rest |
| #8 | spontaneous |
| #9 | #4 or #5 or #6 or #7 or #8 |
| #10 | ALFF |
| #11 | amplitude of low frequency fluctuations |
| #12 | low frequency fluctuations |
| #13 | ReHo |
| #14 | regional homogeneity |
| #15 | local synchronization |
| #16 | ICA |
| #17 | independent component analysis |
| #18 | rCBF |
| #19 | regional cerebral blood flow |
| #20 | rCMRglu |
| #21 | regional cerebral metabolic |
| #22 | glucose metabolism |
| #23 | PET |
| #24 | positron emission tomography |
| #25 | SPECT |
| #26 | single photon emission computed tomography |
| #27 | neural activity |
| #28 | brain activity |
| #29 | #10 or #11 or #12 or #13 or #14 #15 or #16 or #17 or #18 or #19 or #20 or #21 or #22 or #23 or #24 or #25 or #26 or #27 or #28 |
| #30 | #3 and #9 and #29 |

**Supplementary Table 2 Quality assessment checklist[1]**

| **Category 1: Participants** |
| --- |
| 1. Patients were evaluated prospectively, specific diagnostic criteria were applied, and demographic data were reported.  2. Comparison participants were evaluated prospectively psychiatric and medical illnesses were excluded.  3. Important variables (e.g., age, sex, illness duration, onset, medication status, handedness) were checked either by stratification or statistically.  4. Sample size per group > 10. |
| **Category 2: Methods for image acquisition and analysis** |
| 5. Whole brain analysis was automated with no a priori regional selection.  6. Coordinates reported in a standard space.  7. The imaging technique used was clearly described so that it could be reproduced.  8. Measurements were clearly described so that they could be reproduced. |
| **Category 3: Results and conclusions** |
| 9. Statistical parameters for significant and important non-significant differences were provided.  10. Conclusions were consistent with the results obtained and the limitations were discussed. |
| TOTAL /10 |

1. Lan H, Suo X, Li W, Li N, Li J, Peng J, Lei D, Sweeney JA, Kemp GJ, Peng R *et al*: **Abnormalities of intrinsic brain activity in essential tremor: A meta-analysis of resting-state functional imaging**. *Hum Brain Mapp* 2021, **42**(10):3156-3167.

**Supplementary Table 3 Quality assessment of the included studies**

|  | **1** | **2** | **3** | **4** | **5** | **6** | **7** | **8** | **9** | **10** | **Total** |
| --- | --- | --- | --- | --- | --- | --- | --- | --- | --- | --- | --- |
| **Wolf et al. 2014** | 0.5 | 0.5 | 1 | 1 | 1 | 1 | 1 | 1 | 1 | 1 | 9 |
| **Werner et al.2013** | 1 | 0.5 | 1 | 1 | 1 | 1 | 1 | 1 | 0.5 | 1 | 9 |
| **Poudel et al.2014** | 0.5 | 1 | 1 | 1 | 1 | 1 | 1 | 1 | 1 | 1 | 9.5 |
| **Liu et al. 2016** | 1 | 1 | 0.5 | 0.5 | 1 | 1 | 1 | 1 | 0.5 | 1 | 8.5 |
| **Sarappa et al.2017** | 1 | 1 | 1 | 1 | 1 | 1 | 1 | 1 | 0.5 | 1 | 9.5 |
| **Harrington et al.2015** | 1 | 0.5 | 0.5 | 1 | 1 | 1 | 1 | 1 | 1 | 1 | 9 |

**Supplementary Table 4 The subgroup meta-analysis: altered resting-state activity in pHD and sHD patients relative to HCs.**

| **Contrast** | **Brain Regions** | **MNI coordinates** | | | **SDM-Z score** | **No. of voxels** | **p-value** | **Egger's test(p)** | **Clusters' breakdown** | **Jackknife sensitivy analysis** |
| --- | --- | --- | --- | --- | --- | --- | --- | --- | --- | --- |
|  |  | **x** | **y** | **z** |  |  |  |  |  |  |
| **sHD>HC** | Area 1 (Right inferior temporal gyru) | 54 | -20 | -30 | 2.801 | 1316 | <0.001 | 0.378 | Right inferior temporal gyrus | 5/5 |
|  |  |  |  |  |  |  |  |  | Right inferior network, inferior longitudinal fasciculus | 5/5 |
|  |  |  |  |  |  |  |  |  | Right fusiform gyrus | 5/5 |
|  |  |  |  |  |  |  |  |  | Right middle temporal gyrus | 5/5 |
|  |  |  |  |  |  |  |  |  | Corpus callosum | 4/5 |
|  | Area 2 (Corpus callosum) | 12 | 52 | -16 | 1.578 | 375 | 0.003 | 0.987 | Right superior frontal gyrus, medial orbital | 3/5 |
|  |  |  |  |  |  |  |  |  | Right gyrus rectus | 3/5 |
|  |  |  |  |  |  |  |  |  | Corpus callosum | 4/5 |
|  |  |  |  |  |  |  |  |  | Right superior frontal gyrus, orbital part | 3/5 |
|  |  |  |  |  |  |  |  |  | Left superior frontal gyrus, medial orbital | 3/5 |
|  |  |  |  |  |  |  |  |  | Left gyrus rectus | 3/5 |
|  | Area 3 (Right cerebellum, hemispheric lobule VI) | 22 | -72 | -20 | 1.714 | 254 | 0.002 | 0.807 | Right cerebellum, hemispheric lobule VI | 3/5 |
|  |  |  |  |  |  |  |  |  | Right cerebellum, crus I | 3/5 |
| **sHD<HC** | Area 1 (Left superior frontal gyrus, medial) | -6 | 58 | 16 | -2.207 | 435 | <0.001 | 0.029 | Left superior frontal gyrus, medial | 3/5 |
|  |  |  |  |  |  |  |  |  | Right superior frontal gyrus, medial | 4/5 |
|  |  |  |  |  |  |  |  |  | Corpus callosum | 3/5 |
|  |  |  |  |  |  |  |  |  | Left superior frontal gyrus, medial | 3/5 |
|  |  |  |  |  |  |  |  |  | Left anterior cingulate / paracingulate gyri | 3/5 |
|  |  |  |  |  |  |  |  |  | Right anterior cingulate / paracingulate gyri | 3/5 |
|  | Area 2 (Right striatum) | 28 | -6 | -2 | -1.781 | 258 | 0.002 | 0.705 | Right lenticular nucleus, putamen | 3/5 |
|  |  |  |  |  |  |  |  |  | Right striatum | 3/5 |
|  |  |  |  |  |  |  |  |  |  |  |
| **pHD>HC** | Area 1 (Right thalamus) | 10 | -12 | 16 | 1.548 | 202 | <0.001 | 0.244 | Right thalamus | 3/5 |
|  |  |  |  |  |  |  |  |  | Right anterior thalamic projections | 3/5 |
|  |  |  |  |  |  |  |  |  | Right caudate nucleus | 3/5 |
|  |  |  |  |  |  |  |  |  | Corpus callosum | 5/5 |
| **pHD<HC** | Area 1 (Left anterior cingulate / paracingulate gyri) | 0 | 50 | 8 | -1.705 | 279 | <0.001 | 0.557 | Left anterior cingulate / paracingulate gyri | 3/5 |
|  |  |  |  |  |  |  |  |  | Right anterior cingulate / paracingulate gyri | 3/5 |
|  |  |  |  |  |  |  |  |  | Left superior frontal gyrus, medial | 3/5 |
|  | Area 2 (Right anterior thalamic projections) | 4 | -14 | 2 | -1.682 | 102 | 0.001 | 0.732 | Right anterior thalamic projections | 2/5 |

**Abbreviations: pHD: premanifest HD; sHD: symptomatic HD.**

**Supplementary Figure 1 Funnel plots of identified brain regions with increased brain activity in HD patients**

**
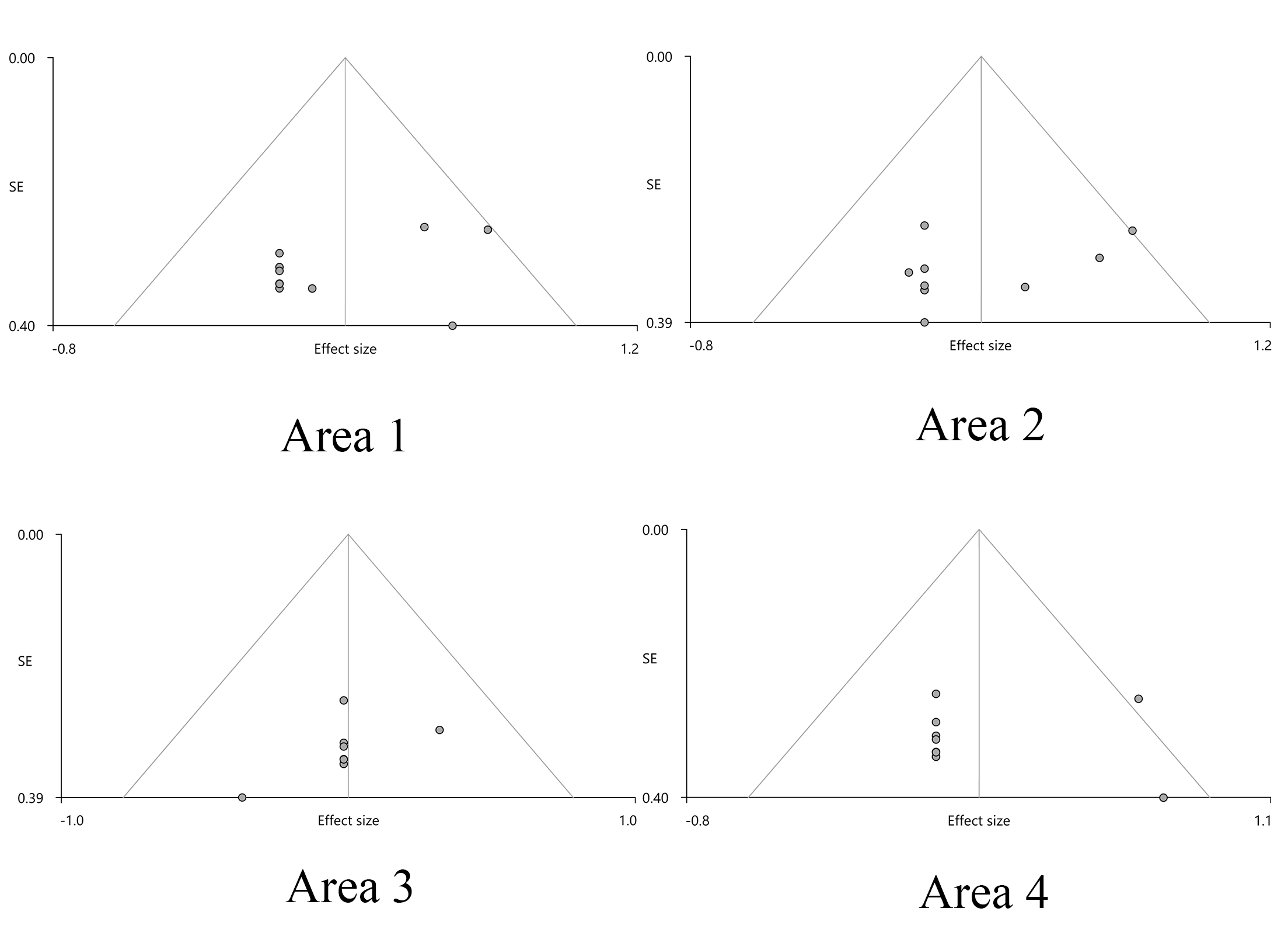
**

**Supplementary Figure 2 Funnel plots of identified brain regions with decreased brain activity in HD patients**

**
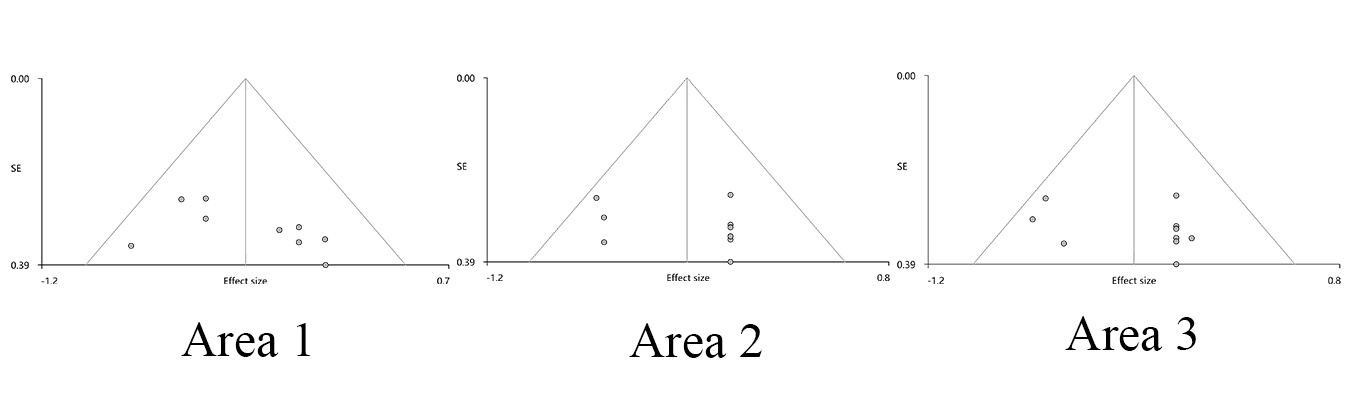
**
